# How Do People Become W.E.I.R.D.? Migration Reveals the Cultural Transmission Mechanisms Underlying Variation in Psychological Processes

**DOI:** 10.1371/journal.pone.0147162

**Published:** 2016-01-13

**Authors:** Alex Mesoudi, Kesson Magid, Delwar Hussain

**Affiliations:** 1 Human Biological and Cultural Evolution Group, Department of Biosciences, University of Exeter Cornwall Campus, Penryn, Cornwall, United Kingdom; 2 Department of Anthropology, Durham University, Durham, United Kingdom; 3 School of Social and Political Science, University of Edinburgh, Edinburgh, United Kingdom; University of Stirling, UNITED KINGDOM

## Abstract

Cultural psychologists have shown that people from Western, Educated, Industrialised, Rich, Democratic (WEIRD) countries often exhibit different psychological processing to people from less-WEIRD countries. The former exhibit more individualistic and less collectivistic social orientation, and more analytic and less holistic cognition, than non-Westerners. Yet the mechanisms responsible for maintaining this cultural variation are unclear. Immigration is an ideal ‘natural experiment’ for uncovering such mechanisms. We used a battery of psychological measures previously shown to vary cross-culturally to compare the social orientation and cognitive style of 286 residents of East London from three cultural backgrounds: (i) 1^st^-generation British Bangladeshi immigrants; (ii) 2^nd^-generation British Bangladeshis raised in the UK to Bangladeshi-raised parents; and (iii) non-migrants whose parents were born and raised in the UK. Model comparison revealed that individualism and dispositional attribution, typical of Western societies, are driven primarily by horizontal cultural transmission (e.g. via mass media), with parents and other family members having little or no effect, while collectivism, social closeness and situational attribution were driven by a mix of vertical/oblique cultural transmission (e.g. via family contact) and horizontal cultural transmission. These individual-level transmission dynamics can explain hitherto puzzling population-level phenomena, such as the partial acculturation of 2^nd^-generation immigrants on measures such as collectivism (due to the mix of vertical and horizontal cultural transmission), or the observation in several countries of increasing individualism (which is transmitted horizontally and therefore rapidly) despite little corresponding change in collectivism (which is transmitted partly vertically and therefore more slowly). Further consideration of cultural transmission mechanisms, in conjunction with the study of migrant communities and model comparison statistics, can shed light on the persistence of, and changes in, culturally-variable psychological processes.

## Introduction

One of the most significant recent developments in psychology has been the documentation of systematic cross-cultural variation in psychological processes that were once thought to be human universals [1–4]. While Westerners typically exhibit an analytic cognitive style, attending more to focal objects and individuals independently of context and using rule-based reasoning, East Asians typically exhibit holistic cognitive style, attending more to contextual relations between objects and individuals and using relationship-based reasoning [5,6]. For example, when presented with object triads such as cow, chicken and grass, Westerners typically group objects according to formal rules (e.g. grouping cow and chicken because they are both members of a “farm animal” category) while East Asians typically group objects according to relationships (e.g. grouping cow and grass, because cows eat grass) [6,7]. This extends to explanations of other people’s behaviour: Westerners typically attribute others’ actions to internal dispositions (e.g. explaining a student’s exam failure in terms of their laziness or lack of intelligence), while East Asians typically attribute others’ actions to external situations (e.g. explaining a student’s exam failure in terms of an unusually difficult exam paper, or because of an overbearing societal pressure to succeed academically) [8]. The over-attribution of actions to internal dispositions, once thought to be a universal error of human cognition [9], turns out to be a peculiarity of Western thinking. Similarly, until recently it was thought that people everywhere show unrealistic self-enhancement: studies conducted in the West show that most people rate themselves as above-average on various desirable qualities such as intelligence, conscientiousness or health [10], even though this is statistically impossible. Yet cross-cultural studies show unrealistic self-enhancement to be much reduced, and often entirely absent, in non-Western societies [11,12]. These differences tap into broader cultural variation in social orientation: East Asians typically possess collectivistic or interdependent selves, defining themselves in terms of social relationships and roles, whereas Westerners typically possess more individualistic or independent selves, describing themselves in terms of intrinsic and individual psychological dispositions [13,14]. Other domains exhibiting cross-cultural variation include fairness norms, moral reasoning, aggression and personality [3]. Consequently, a recent review criticised the traditional focus within psychology on people from Western, educated, industrialised, rich, democratic (“WEIRD”) countries, who seem far from representative of our species as a whole [2].

However, the means by which this cultural variation is maintained over time, i.e. the transmission mechanisms by which different values and thinking styles are passed from person to person, are currently unclear. As one review states, “although much has been learned about cultural differences in behavior and brain responses in recent years, much less is known about how such different responses are learned and acquired” [4] p.441. There is also evidence that culturally variable psychological processes are changing over time, such as increasing individualism in both the US and Japan [15]. Again, however, the individual-level transmission processes that are responsible for this population-level cultural change are unclear.

One possibility is that culturally-variable psychological processes are passed from person to person via cultural transmission (or social learning), with people learning how to, for example, categorise objects or explain others’ actions through observation or instruction, or from the social environments constructed by others. Given that we learn most often from members of our own societies (our parents, our teachers etc.), then this can act to maintain the observed cross-cultural variation. Yet seldom in the cultural psychology literature is large-scale geographic variation in psychological processes explicitly linked to different pathways and mechanisms of cultural transmission at the individual level. In contrast, cultural transmission has been extensively modelled and studied empirically in the cultural evolution literature [16–19], and these models and findings can provide a valuable theoretical framework for explaining the persistence of, and changes in, cultural variation in psychological processes. Cultural evolution researchers distinguish between vertical cultural transmission (learning from one’s parents), oblique cultural transmission (learning from older non-parents, e.g. teachers) and horizontal cultural transmission (learning from peers) [16]. Although parents are popularly thought to constitute a major influence on people’s psychology, there is evidence that horizontal cultural transmission is at least as influential as vertical cultural transmission, if not more so [20]. Horizontal transmission can be divided into several sub-processes [19], including conformity (preferentially adopting majority behaviors), prestige bias (preferentially copying prestigious individuals) and one-to-many transmission (typical of mass media).

Another possibility is that putative ‘cultural’ variation in psychological processes is actually genetic, rather than cultural. Recent research suggests that genetic variation may underlie, or at least influence, some psychological differences [21,22]. For example, East Asian societies may be more collectivistic because these populations have higher frequencies of alleles associated with mood and depressive disorders, and collectivism functions as a social buffer to such disorders [21]. Here, collectivism is suggested to be a cultural response to genetic variation rather than collectivism itself being genetically inherited. Nevertheless, even the latter (genetic variation directly underlying ‘cultural’ variation) remains a possibility sometimes mooted in the literature [22].

Our aim here is to use immigrant populations to test which of these hypothesised transmission mechanisms–horizontal, oblique or vertical cultural transmission, or genetic inheritance—are responsible for the maintenance of cross-cultural variation in psychological processes. While immigrants are not a randomly selected population, immigration is nevertheless an excellent ‘natural experiment’ for this purpose, as it dissociates different influences (e.g. parental and peer psychology) that are confounded in non-migrant populations. If immigrants originating from a non-Western society adopt the local psychological values of their adopted Western society within one or two generations, then a direct genetic explanation can be ruled out. If 1^st^ generation immigrants immediately shift from non-Western to Western values irrespective of age of migration, this indicates powerful horizontal cultural transmission, such as via cultural products within immediate environments [23]. If the 1^st^ generation remain ‘non-Western’ and their 2^nd^ generation children shift completely to local Western values, then we can infer strong horizontal influences (e.g. peer interaction, media exposure) during childhood, and no influence of parents or older family members. A partial shift in the 2^nd^ generation indicates a mix of horizontal and vertical transmission. Moreover, identifying specific correlates of acculturation in migrant populations, such as frequency of family contact or extent of mass media exposure, can reveal the precise mechanisms of transmission within each broad pathway.

A handful of previous studies have directly compared culturally-variable psychological processes in 1^st^ and 2^nd^ generation immigrants, mostly East Asian immigrants in North America, typically finding that 2^nd^ generation immigrants are intermediate between their East Asian-raised parents’ psychological characteristics and those of local European-descended Westerners on measures including reasoning style [24] and self-enhancement [11]. This substantial but incomplete acculturation in 2^nd^ generation Asian Americans counts against a direct genetic explanation, and suggests a combined influence of vertical and horizontal cultural transmission. However, these studies are limited in that (i) they, like most previous non-immigrant studies, compared East Asian and North American societies, and these findings should be replicated in other Western and non-Western populations; (ii) only a single measure was obtained in each study, so it is unknown whether these acculturation patterns apply just to these specific measures (self-enhancement, categorisation) in these specific participant samples or globally across multiple tasks that tap broad theoretical constructs such as individualism/collectivism or analytic-holistic cognition; and (iii) no potential correlates of acculturation were measured, such as media exposure, education or family contact, that might delineate transmission mechanisms.

Consequently, the present study examined the acculturation of British Bangladeshis in East London, i.e. South Asian immigrants in Western Europe, therefore providing an important complement to previous studies of East Asian immigrants in North America. We administered a battery of measures previously shown to vary cross-culturally, rather than relying on just a single measure, and we measured several potential indicators of cultural transmission mechanisms, to provide better understanding of the precise pathways by which psychological processes are acquired and transmitted. We also employed information-theoretic model selection techniques [25], common in ecology but novel in cultural psychology, that allow different potential models of transmission to be assessed without the limitations of null-hypothesis significance testing [26].

British Bangladeshis were chosen because their population size, migration history and cultural cohesion relative to the rest of London and the UK provide many parallels to the position of East Asian immigrants in North America, yet with interesting differences in aspects such as religious affiliation and history of colonialism. Historically, what is now Bangladesh was under British rule from the mid-1700s until partition in 1947 along religious lines, when due to its Muslim majority it was joined to Pakistan and named East Bengal (later East Pakistan). Modern Bangladesh achieved independence from Pakistan in 1971 following a bloody conflict, and since then has experienced periods of parliamentary democracy interspersed with military rule. Migration from Bangladesh to the UK began predominantly in the 1970s following the war for independence as well as changes in British immigration laws, and migration occurred mostly from the Sylhet region of north-eastern Bangladesh. Today, British Bangladeshis are a geographically concentrated and culturally cohesive migrant community that is more ethnically segregated than other South Asian minorities and the British population as a whole [27]. The most recent (2011) census data show 222,127 people of Bangladeshi descent residing in London constituting 2.7% of the population of London as a whole, although British Bangladeshis make up 32% of the East London borough of Tower Hamlets where this study was conducted [28]. The older 1^st^-generation who migrated in the 1970s often identify strongly with Bangladesh and speak Bengali or Sylheti (a dialect of, or sister language to, Bengali) as a first language, while 2^nd^ and subsequent generations more often identify as British, British Bangladeshi or British Muslim, and speak English as a first language [27]. All generations predominantly identify as Muslim, and indeed 2^nd^ and 3^rd^ generations often prioritise their Muslim identities over their Bangladeshi identities [29]. Historically, British Bangladeshis have experienced economic and social deprivation in the UK, although this has improved in recent years [27]. Taking these population characteristics in their entirety, British Bangladeshis provide a good example of a community originating in a less-WEIRD society (i.e. coming from a non-Western country that, compared to Western countries, has historically had relatively less formal education, less industrialisation, lower wealth, and less democracy—albeit all of these enforced or caused by Western colonialism) but who have lived for multiple generations in a much more WEIRD society (the UK). Note that, as in [2], we view ‘WEIRD-ness’ as a continuum (or rather, multiple continuums) rather than a dichotomy, with people and societies more or less WEIRD rather than ‘WEIRD’ or ‘non-WEIRD’.

We administered eight measures of cognitive style and social orientation previously shown to vary cross-culturally (individualism, collectivism, social closeness, self-enhancement, holistic/analytic categorisation, dispositional/situational attribution, and analytic/holistic drawing style) to (i) 1^st^-generation British Bangladeshi immigrants born and raised in Bangladesh who moved to the UK after the age of 14 (henceforth ‘1^st^ generation’ or ‘1^st^ gen’), (ii) 2^nd^-generation British Bangladeshis born in the UK to 1st-generation British Bangladeshis (henceforth ‘2^nd^ generation’ or ‘2^nd^ gen’), and (iii) non-migrant residents of the same area of London whose parents, like themselves, were born and raised in the UK (henceforth ‘non-migrants’). Each group contained a wide range of ages and socio-economic classes, going beyond typical student samples. A cut-off of 14 years was used given previous findings of a 14-year acculturation sensitive period [30].

Little past research has applied these measures to South Asian societies specifically. A meta-analysis of individualism-collectivism [31] included no studies for Bangladesh, although it did include one study [32] that reported data for Pakistan, probably the country with the strongest historical, social and religious similarities to Bangladesh. This study found much higher collectivism in Pakistan compared to English-speaking countries, and slightly (but non-significantly) lower individualism [32]. Given that collectivistic countries tend to show more holistic cognition [33], we predict that our 1^st^ generation British Bangladeshi participants will resemble other non-Western (e.g. East Asian) respondents and exhibit more collectivistic and less individualistic social orientation, and more holistic and less analytic cognition, than non-migrant participants. As noted above, 2^nd^ generation participants represent a crucial test of the relative role of vertical and horizontal transmission. If they are identical to the 1^st^ generation, then cultural variation is maintained by strong vertical genetic or cultural transmission. If they resemble non-migrants, then horizontal cultural transmission plays a major role. In addition, we measured various individual characteristics that may serve as specific drivers of cognitive change, such as family contact, media use, religiosity, education and socio-economic status.

## Materials and Methods

### Participants

Participants were all residents of Greater London and recruited within Tower Hamlets, East London via local schools/colleges, community groups and personal contacts from Jan 2012—Dec 2014 (see Table 1 for full demographic information). Each participant was compensated with £5 and provided written consent. The study was approved by Durham University Department of Anthropology’s Ethics Review Board. There were 330 participants in total, although 44 were excluded due to unsuitable cultural background (not having spent their first 14 years in Bangladesh or the UK, or not having two UK-born or two Bangladeshi-born parents). Of the remaining 286, 144 were female. 99 were non-migrants, born and raised in the UK to UK-born and raised parents. 108 were 1^st^ generation British Bangladeshi, born and raised in Bangladesh at least until age 14, before moving to the UK at least one year prior to participation. 79 were 2^nd^ generation British Bangladeshi, born and raised in the UK (or, in 5 cases, born in Bangladesh but moved to the UK before age 14) to parents both of whom can be classified as 1^st^ generation. We did not record or target actual parent-child relationships. Roughly half of each group was female. The mean and spread of ages for non-migrants and the 1^st^ generation were comparable, while the 2^nd^ generation were slightly younger due to the relatively recent Bangladeshi-UK migration. 1^st^ and 2^nd^ generation British Bangladeshis had larger family networks, higher religiosity, and spoke more languages (typically English and Bengali) than non-migrants. The 1^st^ generation had slightly less education, lower occupational socio-economic status (SES), and less exposure to UK-based mass media than non-migrants and the 2^nd^ generation. Finally, the self-report acculturation measures showed that 1^st^ generation British Bangladeshis identify more strongly with their heritage (i.e. Bengali) culture than the 2^nd^ generation, while the 2^nd^ generation identify more strongly with UK mainstream culture than the 1^st^-generation.

**Table 1:** Participant demographics.

	Non-migrant	2^nd^-generation British Bangladeshi	1^st^-generation British Bangladeshi	All
n (n female)	99 (50)	79 (40)	108 (54)	286 (144)
Age, mean (sd, range)	35.07 (15.11, 18–73)	26.09 (7.91, 18–52)	39.48 (12.13, 19–75)	34.26 (13.41, 18–75)
Religiosity (min = 1, max = 7), mean (sd)	1.86 (1.21)	4.10 (1.44)	4.83 (1.34)	3.60 (1.85)
Family contact, mean (sd)	3.09 (2.44)	7.11 (4.83)	7.33 (5.23)	5.73 (4.72)
Family interaction, mean (sd)	2.61 (2.04)	6.39 (5.90)	6.79 (6.05)	5.09 (5.25)
Years of education, mean (sd)	16.31 (3.96)	16.90 (4.07)	14.28 (6.04)	15.71 (4.99)
Occupation, n (secondary / tertiary / graduate)	26 / 37 / 16	21 / 31 / 15	33 / 28 / 7	80 / 96 / 38
Languages spoken, mean (sd)	1.12 (0.39)	2.01 (0.44)	2.13 (0.95)	1.75 (0.81)
UK TV watched per week (hours), mean (sd)	3.07 (2.26)	3.51 (2.38)	2.66 (1.52)	3.03 (2.06)
Internet use per week (hours), mean (sd)	3.26 (1.51)	4.12 (2.91)	2.72 (1.88)	3.29 (2.17)
UK print media use (min = 1, max = 4), mean (sd)	2.70 (0.77)	2.50 (0.82)	2.19 (0.82)	2.45 (0.83)
Heritage culture identification (min = 1, max = 7), mean (sd)	N/A	5.32 (1.07)	5.85 (1.03)	5.60 (1.07)
UK culture identification (min = 1, max = 7), mean (sd)	N/A	5.27 (1.08)	4.51 (1.36)	4.83 (1.29)
Age of migration, mean (sd)	N/A	N/A	21.57 (6.60)	N/A

**Notes:** ‘Family contact’ = number of family members seen in person during an average week; ‘Family interaction’ = number of family members communicated by phone/internet during an average week. ‘Occupation’ is categorised into level of education needed: secondary (school leaving exams required), tertiary (university degree required) or graduate (post-university qualification required, e.g. Masters). Self-reported acculturation measures (heritage and UK culture identification) were only obtained from the British Bangladeshi groups, and age of migration only applies to the 1^st^ generation group.

### Materials

All participants completed the same experimental booklet containing six parts followed by demographic information (see Table 2 for details of the measures, and S1 File for the full experimental booklet). Part 1 comprised an individualism-collectivism questionnaire [14] containing eight items measuring individualism and eight measuring collectivism on 7-point Likert scales. Following [31], we analysed individualism and collectivism as separate constructs, rather than opposite ends of a continuum. Part 2 measured social closeness using the Inclusion of Other in the Self (IoS) scale [34]: participants chose circles of varying overlap to indicate closeness to their most significant other. Part 3 measured self-enhancement: participants estimated the percentage of the UK population of the same age and gender who exceed them on 10 desirable characteristics (e.g. intelligence, attractiveness) [12]. Part 4 measured holistic/analytic categorisation: for 10 triads of objects (e.g. horse, goat, saddle), participants circled two that go together [7]; holistic responses indicate relationships (e.g. horse-saddle), analytic responses indicate rule-based similarity (e.g. horse-goat). Part 5 measured social attribution [8]: participants read two stories about an individual’s actions (e.g. Ben Johnson’s Olympics cheating) and rated their agreement on 7-point Likert scales with dispositional (e.g. “Johnson took steroids because of his excessive drive to win”) and situational (e.g. “Johnson took steroids because athletics had become too competitive”) explanations. In Part 6 participants drew a landscape scene [35]; previous findings suggest that holistic thinkers draw more additional objects and higher horizons to accommodate more object inter-relationships, compared to analytic thinkers. A final section asked various demographic and lifestyle questions: age, gender, occupation, parents’ occupations, years of formal education, highest qualification, languages spoken fluently, number of family members seen in person / contacted per week, use of UK print media, UK TV, and the internet, religiosity, (for British Bangladeshis only) self-reported heritage/mainstream acculturation using the Vancouver Index of Acculturation [36], and (for 1^st^ generation British Bangladeshis only) age of migration.

**Table 2:** Tasks used in the present study, and previously-found cultural differences.

Measure	Description	Previous findings	Key reference
**Social orientation**	Participants are asked their agreement on 7-point Likert scales with 16 statements indicative of individualism and collectivism.	English-speaking countries are typically more individualistic and less collectivistic than the rest of the world.	[14]
**Inclusion of Other in the Self**	Participants choose one of 7 pairs of more or less overlapping circles that best describes themselves and their most significant other.	East Asians typically choose more-overlapping circles than North Americans, indicating higher social closeness.	[34]
**Self-enhancement**	Participants estimate the percentage of the UK population, of the same age and gender, who are better than them on 10 desirable characteristics (e.g. attractiveness, intelligence).	North Americans typically show higher or unrealistically biased self-enhancement, with most participants rating themselves above-average, compared to East Asians.	[12]
**Categorisation**	Participants circle two objects that go together within a series of 10 triads (e.g. horse, saddle, goat).	North Americans typically use rule-based similarity, e.g. grouping horse and goat (as both are farm animals), while East Asians typically use relationships, e.g. grouping horse and saddle (as horses wear saddles)	[7]
**Social attribution**	Participants read descriptions of two real-life events (Ben Johnson cheating in the Olympics; a physics student shooting his supervisor) and rate agreement on 7-point Likert scales with various explanations.	North Americans typically agree more with dispositional explanations (e.g. “Johnson took steroids because of his excessive drive to win”) and less with situational attributions (e.g.“Johnson took steroids because athletics had become too competitive”) than East Asians.	[8]
**Drawing task**	Participants draw a landscape scene, including a house, tree, river, person, horizon and any other additional objects	North Americans typically draw fewer additional objects and a lower horizon, given a focus on fewer, focal objects and simple scenes (analytic cognition), while East Asians typically include many objects and high horizons to display their interconnections (holistic cognition)	[35]

### Analyses

Analyses were conducted in R version 3.2.2 [37]. Analysis proceeded in three stages (see S2 File for full data set and S3 File for data R analysis scripts). To first assess basic group differences in each measure, we ran regression models with cultural group (1^st^ generation, 2^nd^ generation or non-migrant) as the sole predictor, followed by Tukey post-hoc tests using package *multcomp* [38]. We present 95% confidence intervals rather than p-values following recent calls to move away from dichotomous (significant / non-significant) null-hypothesis testing [26]. Linear regression was used for individualism and collectivism (following reflection and log transformation due to negative skew), and self-enhancement and dispositional/situational attribution (untransformed). For closeness we ran ordinal logistic regression using clm from package *ordinal* [39]. Categorisation (holistic responses divided by total holistic and analytic responses) was an under-dispersed proportion so quasibinomial regression was used. The number of additional objects drawn in the drawing task was a count variable with large variance relative to the mean so negative binomial regression was run using package *MASS* [40].

Second, we used model comparison techniques [25] to compare the fit to the data of alternative models that stem from the hypothesised transmission pathways outlined above. Due to the lack of previous quantitative model-fitting in this field, models omitted interactions to keep the model set as small as possible. For each measure, seven models were compared (Table 3). A demographic model (DEMOGRAPHIC) contained only age and sex, with no cultural indicators. PARENTSBIRTH contained age, sex and parents’ country of birth, representing strong parental influence via vertical cultural transmission or genetic inheritance. COUNTRYBIRTH contained age, sex and participant’s country of birth, representing strong horizontal transmission during a developmental sensitive period. CULTURALGROUP contained age, sex, participant’s and parents’ country of birth, and delineates the three cultural groups (1^st^-generation, 2^nd^-generation and non-migrants) examined previously. HORIZONTAL contained age, sex, plus all predictors associated with horizontal cultural transmission: participant’s country of birth, UK print media use, internet use, UK TV use, and years of education. Note that the print media were specifically designated in the experimental materials as UK-based, justifying their inclusion as markers of horizontal cultural transmission. Internet use was not designated as such, but we have independent evidence that British Bangladeshis seldom access Bangladeshi websites [27], p.55. VERTICAL contained age, sex, plus all predictors associated with vertical or oblique (i.e. within Bangladeshi families) cultural transmission: parents’ country of birth, family direct contact, family indirect interaction, heritage language fluency (a dichotomous measure indicating whether the participant speaks Bengali fluently or not) and religiosity. Finally, a global model (GLOBAL) contained all aforementioned predictors, and was used to assess overall suitability of model comparison [25], p.26. MuMIn [41] was used to obtain model fit statistics.

**Table 3:** Summary of predicted models used in model comparison.

Model	Predictors
DEMOGRAPHIC	Age, sex
PARENTSBIRTH	Age, sex, country of parents’ birth (UK or Bangladesh)
COUNTRYBIRTH	Age, sex, country of participant’s birth (UK or Bangladesh)
CULTURALGROUP	Age, sex, country of parents’ birth, country of participant’s birth
HORIZONTAL	Age, sex, country of participant’s birth, UK print media use, internet use, UK TV use, years of education
VERTICAL	Age, sex, country of parents’ birth, family direct contact, family indirect interaction, heritage language fluency, religiosity.
GLOBAL	All predictors listed above

The third analysis stage was explicitly exploratory using stepwise regression to explore the significance of individual predictors and potential interactions for each measure. While acknowledging the dangers of data dredging [25], this was conducted to provide future studies with specific quantitative models to compare in new datasets. This also allowed us to explore variables for which we had only partial data: the acculturation measures which only applied to the British Bangladeshi participants, age of migration which only applied to the 1^st^ generation British Bangladeshis, and occupational socio-economic status (SES), which was only obtainable for some participants due to partial or ambiguous responses to participants’ and their parents’ occupations. Occupational SES is considered a marker of horizontal cultural transmission given that it indicates extent of participation in a Western market economy [42]. Also included in the exploratory regressions was number of languages spoken, in addition to heritage language fluency.

## Results

**Fig 1:**
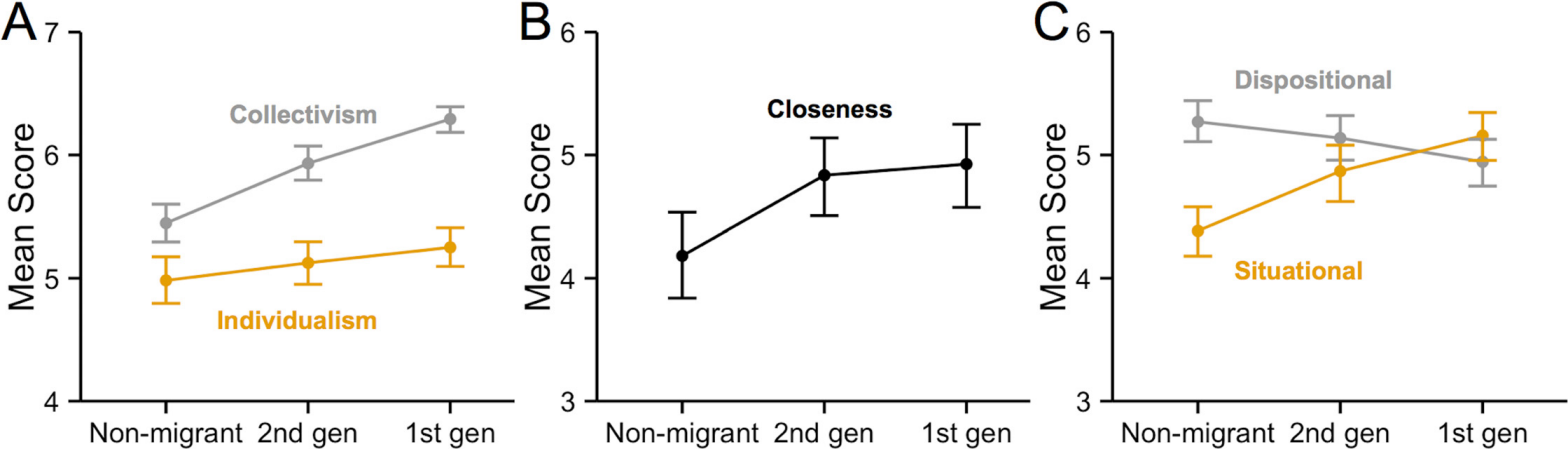
Cultural group differences indicated as meaningful by the culture-only regression analyses, for (A) social orientation, (B) closeness to a significant other, and (C) dispositional/situational attribution. Error bars show 95% confidence intervals. 1^st^ gen = 1^st^ generation British Bangladeshi, 2^nd^ gen = 2^nd^ generation British Bangladeshi.

The culture-only regressions showed different effects of cultural group for the different measures (Table 4). 1^st^ generation British Bangladeshis were more collectivistic than non-migrants, with the 2^nd^ generation intermediate between the 1^st^ generation and non-migrants (Fig 1A). The 1^st^ generation also showed slightly higher individualism than non-migrants, with the 2^nd^ generation again intermediate, although this effect was much weaker. 1^st^ and 2^nd^ generation British Bangladeshis showed similar social closeness, and both groups were much higher in closeness than non-migrants (Fig 1B). For attribution, 1^st^ generation British Bangladeshis were less dispositional and more situational than non-migrants (Fig 1C). The 2^nd^ generation grouped with the 1^st^ generation for situational, and with non-migrants for dispositional. Self-enhancement, categorisation and the drawing task showed no cultural group differences.

**Table 4:** Summary of culture-only means and regression coefficients. Means and standard deviations are raw values before transformation. Unstandardised regression coefficients estimate the difference denoted in the column heading, with 95% confidence intervals in square brackets. Note that in the regressions several measures are logged, and some models are non-linear (see text for details), so coefficients should not be compared across models/measures. Differences comprising CIs that do not cross zero are shown in bold. 1^st^ gen = 1^st^ generation British Bangladeshi, 2^nd^ gen = 2^nd^ generation British BangladeshiFor categorisation, higher values indicate holistic cognition, lower indicate analytic.

Measure	Mean (sd), before transformation			Unstandardised regression coefficients [95% CI] on group differences		
	1^st^ gen	2^nd^ gen	Non-migrants	1^st^ gen - 2^nd^ gen	1^st^ gen–non-migrants	2^nd^ gen–non-migrants
Individualism	5.25 (0.82)	5.13 (0.83)	4.98 (0.95)	0.05 [-0.06, 0.15]	0.08 [-0.02, 0.19]	0.04 [-0.07, 0.15]
Collectivism	6.29 (0.57)	5.93 (0.66)	5.45 (0.77)	**0.19 [0.08, 0.30]**	**0.41 [0.30, 0.51]**	**0.22 [0.11, 0.33]**
Closeness	4.18 (1.79)	4.84 (1.44)	4.93 (1.87)	0.19 [-0.42, 0.79]	**0.80 [0.21, 1.40]**	**0.62 [0.01, 1.23]**
Self-enhancement	37.92 (13.97)	40.86 (17.70)	38.14 (15.48)	-2.72 [-8.22, 2.77]	0.22 [-4.95, 5.38]	2.94 [-2.65, 8.53]
Categorisation (holistic)	0.83 (0.27)	0.82 (0.27)	0.76 (0.29)	0.06 [-0.58, 0.69]	0.41 [-0.15, 0.98]	0.36 [-0.26, 0.97]
Dispositional attribution	4.94 (1.01)	5.14 (0.79)	5.27 (0.88)	-0.19 [-0.51, 0.13]	**-0.33 [-0.62, -0.03]**	-0.13 [-0.46, 0.19]
Situational attribution	5.16 (1.08)	4.87 (1.04)	4.38 (1.01)	0.29 [-0.08, 0.65]	**0.77 [0.43, 1.12]**	**0.48 [0.11, 0.86]**
Drawing task: Horizon ratio	0.59 (0.22)	0.59 (0.18)	0.60 (0.18)	0.01 [-0.07, 0.08]	-0.004 [-0.07, 0.06]	-0.01 [-0.09, 0.06]
Drawing task: Objects	15.43 (24.95)	11.91 (11.39)	14.38 (18.98)	0.02 [-0.36, 0.41]	-0.03 [-0.40, 0.33]	-0.06 [-0.45, 0.33]

Model comparison revealed different transmission pathways for different measures (Table 5, see S1 Table for full model statistics). For individualism, HORIZONTAL was clearly supported, with no other models within the recommended AIC_c_ difference (∆_i_) of 4 [25]. In contrast, CULTURALGROUP was the best-supported model for collectivism, along with GLOBAL and VERTICAL, indicating a mix of parental and non-parental influence. Multimodel inference [25] showed that participant’s country of birth (0.17, 95% CI[0.04, 0.28]) and sex (-0.09, 95% CI[-0.18, -0.01] were the most important predictors of collectivism across these three best-supported models, with family contact (0.01, 95% CI[0.00, 0.02]) and religiosity (0.03, 95% CI[0.00, 0.06]) also important. Closeness was predicted mostly by vertical/parental models, with PARENTSBIRTH, VERTICAL and CULTURALGROUP all showing support. Multimodel inference showed that family interaction (0.048, 95% CI[0.00, 0.09]) and country of parents’ birth (0.74, 95% CI[-0.08, 1.55]) were the most important predictors of closeness across these three best-supported models. The two attribution measures showed broadly mirror effects based on models incorporating place of birth variables: COUNTRYBIRTH and CULTURALGROUP best determined dispositional, while PARENTSBIRTH and CULTURALGROUP best determined situational. VERTICAL, PARENTSBIRTH and HORIZONTAL also fell within the ∆_i_<4 cutoff for dispositional attribution, although with relatively weak support (∆_i_ of around 2 and above). Consistent with this, multi-model inference showed that country of participant’s birth (-0.35, 95% CI[-0.62, -0.07]) was the most important predictor of dispositional attribution across these five best-supported models, followed by age (0.01, 95% CI[0.00, 0.02]). Conversely, multi-model inference for situational attribution showed country of parents’ birth to be the most important predictor (0.57, 95% CI[0.22, 0.91]), followed again by age (0.01, 95% CI[0.00, 0.02]). Self-enhancement, categorisation and the two drawing task measures (horizon ratio and number of additional objects) all showed poor global model fit, making them unsuitable for multi-model comparison.

**Table 5:** Best-fitting predicted models. ∆_i_ = difference in AIC_c_ from best-fitting model; ω_i_ = Akaike weight. See text and Table 3 for model specifications. All models with ∆_i_<4 are listed. ∆_i_ for best-fitting models is always 0 and so not shown. For four measures (self-enhancement, categorisation, horizon ratio and additional objects), the global model did not fit the data so model comparison was not possible. See S1 Table for full model comparison statistics.

Measure	Best fitting models (where ∆_i_ < 4)
Individualism	HORIZONTAL (ω_i_ = 0.80)
Collectivism	CULTURALGROUP (ω_i_ = 0.58), GLOBAL (ω_i_ = 0.25, ∆_i_ = 1.70), VERTICAL (ω_i_ = 0.16, ∆_i_ = 2.57)
Closeness	PARENTSBIRTH (ω_i_ = 0.47), VERTICAL (ω_i_ = 0.27, ∆_i_ = 1.08), CULTURALGROUP (ω_i_ = 0.18, ∆_i_ = 1.90)
Self-enhancement	Poor global model fit
Categorisation	Poor global model fit
Dispositional attribution	COUNTRYBIRTH (ω_i_ = 0.41), CULTURALGROUP (ω_i_ = 0.23, ∆_i_ = 1.20), VERTICAL (ω_i_ = 0.16, ∆_i_ = 1.90), PARENTSBIRTH (ω_i_ = 0.12, ∆_i_ = 2.51), HORIZONTAL (ω_i_ = 0.06, ∆_i_ = 3.86)
Situational attribution	CULTURALGROUP (ω_i_ = 0.53), PARENTSBIRTH (ω_i_ = 0.43, ∆_i_ = 0.40)
Drawing task: Horizon ratio	Poor global model fit
Drawing task: Objects	Poor global model fit

**Fig 2:**
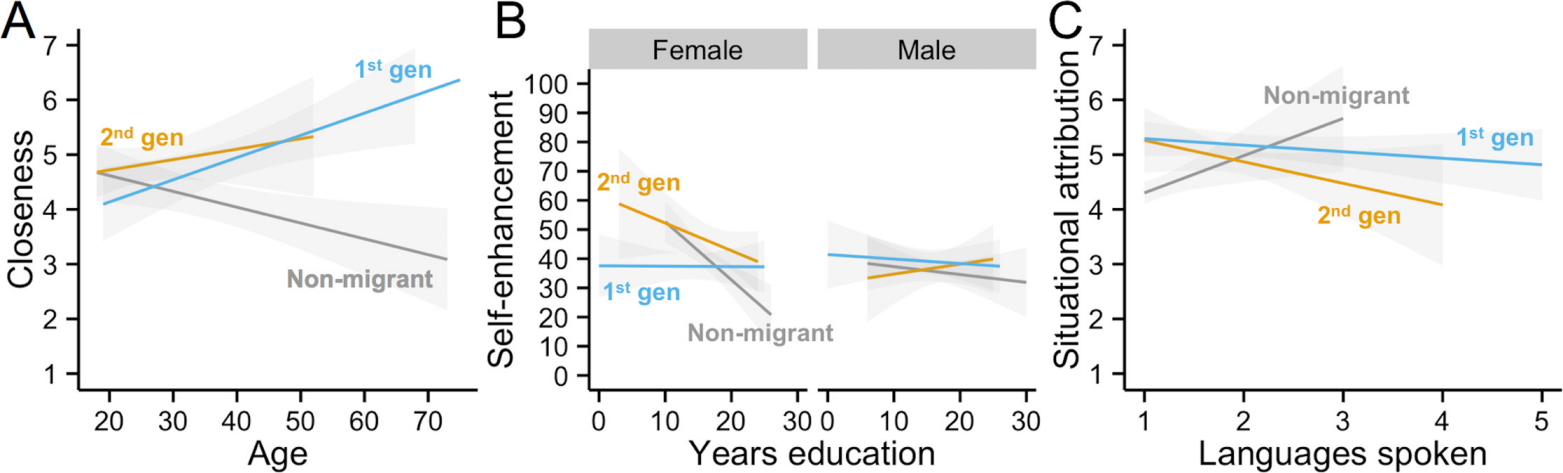
Significant interactions that emerged from the exploratory regression analyses, for (A) closeness, (B) self-enhancement and (C) situational attribution. Shaded areas show conditional means.

Exploratory regression analyses revealed significant predictors for each measure that were broadly consistent with the model comparison, but with some additional interesting interactions (Table 6, see S2 Table for full model statistics). For individualism, the finding that 1^st^ generation British Bangladeshis were more individualistic than non-migrants was repeated, with the 2^nd^ generation non-significantly intermediate; additional significant predictors were occupational SES and UK print media use, both markers of horizontal cultural transmission, consistent with the model comparison. For collectivism, 1^st^ generation British Bangladeshis were more collectivistic than both the 2^nd^ generation and non-migrants, who did not differ. Women were more collectivistic than men, and collectivism increased with religiosity and family contact, both markers of vertical cultural transmission. Closeness showed a significant cultural group x age interaction: non-migrants became less close with age, and 1^st^ and 2^nd^ generation British Bangladeshis became closer (Fig 2A). Self-enhancement showed a three-way interaction between cultural group, sex and years of education (Fig 2B). Men of all cultural backgrounds and education self-enhanced, falling below the 50% ‘realistic’ threshold. 1^st^ generation women also consistently self-enhanced. Non-migrant and 2^nd^ generation women, however, showed increasing self-enhancement with education; the more years of education, the more likely they were to rate themselves better than average. For categorisation, the 1^st^ generation were more holistic (less analytic) than non-migrants, and holistic categorisation decreased (analytic increased) with increasing occupational SES and UK TV viewing. Dispositional attribution increased with age and occupational SES, with no effect of cultural group after accounting for these predictors. Situational attribution showed a cultural group x languages interaction (Fig 2C): for 1^st^ and 2^nd^ generation British Bangladeshis, speaking more languages significantly decreased situational attribution; for non-migrants, speaking more languages significantly increased situational attribution (t = 2.64, p = .001). In the drawing task, neither horizon height nor number of additional objects had any significant predictors.

**Table 6:** Summary of exploratory regression models.

Measure	Predictors in best-fitting model	Model fit
Individualism	Cultural group: 1^st^ gen > non-migrant**; Occupation: graduate > school-leaver*; Print media use***	F(5,201) = 6.99, p < .001, adj-R^2^ = .13
Collectivism	Cultural group: 1^st^ gen > non-migrant***, 1^st^ gen > 2^nd^ gen***; Sex: female > male*; Religiosity*; Family contact*	F(5,262) = 21.11, p < .001, adj-R^2^ = .27
Closeness	2^nd^ gen x age*; 1^st^ gen x age***	Χ^2^(5) = 29.55, p < .001, pseudo-R^2^ = .03
Self-enhancement	1^st^ gen x female x years of education*	F(11,268) = 2.58, p = .004, adj-R^2^ = .06
Holistic categorisation	Cultural group: 1^st^ gen > non-migrant*; Occupation: graduate < school-leaver*; UK TV viewing*	F(5,202) = 3.62, p = .004, pseudo-R^2^ = .08
Dispositional attribution	Age*; Occupation: graduate > school-leaver*	F(5,207) = 3.12, p = .009, adj-R^2^ = .05
Situational attribution	2^nd^ gen x languages**; 1^st^ gen x languages**	F(5,279) = 7.79, p < .001, adj-R^2^ = .11
Drawing task: Horizon ratio	No significant predictors	N/A
Drawing task: Objects	No significant predictors	N/A

*p < .05

**p < .01

***p < .001

Where best-fitting model includes interactions, only highest-level significant interactions are listed, not single predictors (even if significant). ‘languages’ = number of languages spoken. 1^st^ gen = 1st generation British Bangladeshi, 2^nd^ gen = 2nd generation British Bangladeshi. See S2 Table for full model details.

Additional exploratory regression analyses were run for the 1^st^ and 2^nd^ British Bangladeshi groups including UK/heritage acculturation, and for the 1^st^ generation including age of migration, alongside previously-significant predictors for each measure (see S2 Table). Individualism increased with heritage culture (i.e. Bengali) identification, and decreased with UK culture identification, corroborating the finding that 1^st^ generation British Bangladeshis are more individualist than non-migrants. Collectivism also increased with heritage culture identification, corroborating the finding of higher collectivism in the 1^st^ generation, and also showed an effect of age of migration: 1^st^ generation participants who migrated at older ages were more collectivistic, suggesting a longer developmental sensitive period than 14 years. No other measure showed effects of acculturation or age of migration.

## Discussion

Despite the extensive documentation of cultural variation in human psychological processes, little research has examined the precise mechanisms by which this variation is transmitted, maintained and potentially transformed over time. Here we used the natural experiment of migration to begin to uncover these mechanisms. A battery of measures previously shown to vary cross-culturally were administered to two generations of British South Asian (Bangladeshi) residents of East London, UK, along with non-migrant residents of the same area. Based on previous findings with similar South Asian populations [32], we predicted that our 1^st^ generation British Bangladeshi participants, who grew up in a non-Western (or relatively less ‘WEIRD’: [2]) country, would show psychological attributes typical of other non-Western societies, while our British respondents would be typically Western (or relatively more ‘WEIRD’). Our 2^nd^ generation British Bangladeshi participants provide a crucial test of which transmission mechanisms drive a shift from ‘non-Western’ (or less WEIRD) to ‘Western’ (or more WEIRD) thinking styles: the extent to which they resemble their 1^st^ generation parents indicates the strength of vertical or familial cultural transmission (or possibly genetic inheritance), while the extent to which they resemble their non-migrant peers indicates the strength of horizontal cultural transmission.

Several measures showed the predicted ‘Western’ vs. ‘non-Western’ differences between the 1^st^ generation and the non-migrants. 1^st^ generation British Bangladeshis were more collectivistic, showed increased social closeness to others, and showed more situational and less dispositional social attribution than non-migrants, replicating past research with other South and East Asian populations. As expected given past research on South Asian populations in particular [32], individualism showed less variation; in fact the 1^st^ generation were slightly more individualistic than the non-migrants, albeit very weakly. Three measures–self-enhancement, categorisation and drawing style–failed to show any cultural group differences, perhaps suggesting that these dimensions do not vary between Western European and South Asian populations, and instead may be specific to the North American and/or East Asian populations studied in most previous research in cultural psychology.

The finding that the 1^st^ generation British Bangladeshis retained the non-Western social orientation, attributional style and social closeness of their region of origin despite living in the UK, often for decades, counts against any immediate and wholesale effect of the social environment, and instead supports the notion of a developmental sensitive period of up to 14 years during which culturally variable psychological attributes become set [30] (although note that collectivism showed a small decline with time spent in the UK, indicating that this sensitive period is not absolute). Our main comparison of interest, however, was between the 2^nd^ generation and the other groups. For the measures that showed cultural differences (collectivism, individualism, and dispositional and situational attribution), the 2^nd^ generation were typically intermediate between non-migrants and the 1^st^ generation (Fig 1), replicating previous findings [11,24]. This counts against a direct genetic explanation, and suggests a mix of vertical and horizontal cultural transmission that shifts migrants towards the psychological characteristics of their adopted society within one generation. That we see this effect in 2^nd^ generation British Bangladeshis who retain the extensive family ties, strong religious beliefs and fluent heritage language (Bengali) of their parents (see Table 1) attests to the strength of horizontal cultural transmission [20]. An exception to this pattern was closeness, for which the 2^nd^ generation clearly grouped with the 1^st^ generation (Fig 1B). Closeness, which focuses on a single closest other, might be more directly influenced by the more frequent family interactions shown in both British Bangladeshi groups (Table 1).

A more robust test of transmission mechanisms was conducted using model comparison techniques [25], going beyond simple cultural group differences and taking into account multiple additional predictors of transmission. Model comparison revealed that individualism was determined solely by indicators of horizontal cultural transmission, with no evidence of any parental/family influence. In contrast, collectivism was determined by a mix of indicators of both vertical and horizontal cultural transmission: the best supported model (CULTURALGROUP) contained both parents’ country of birth (a marker of vertical cultural transmission) and participant’s country of birth (a marker of horizontal cultural transmission), although a vertical cultural transmission model (VERTICAL) also received support. Multimodel inference showed that participant’s country of birth was the most important predictor of collectivism across all supported models, along with markers of vertical cultural transmission (family contact and religiosity). This mix of vertical and horizontal cultural transmission may explain why 2^nd^ generation British Bangladeshis were intermediate to the other groups in collectivism (Fig 1A), rather than identical to either the 1^st^ generation or to the non-migrants.

These general dynamics may account for hitherto unexplained patterns of cultural change in social orientation. Cultural evolutionary theory predicts that horizontal transmission results in faster population-level cultural change than vertical transmission [16]. Interestingly, several countries are currently increasing in individualism while not changing in collectivism, with the latter stasis seemingly unexplained (e.g. “[p]erhaps the most intriguing aspect of this research is the persistence of collectivism in Japan” [15], p.16). Our findings suggest this is because collectivistic and individualistic values are transmitted through different pathways: the former with a substantial vertical component, thus changing more slowly over time, the latter almost entirely horizontally, thus changing rapidly, particularly when societies are exposed to changing economic or social conditions. These transmission dynamics also make sense in terms of cultural selection: collectivism places high value on parents and family (e.g. values such as “Parents and children must stay together as much as possible”) so it is understandable that parents/families are motivated to transmit such values.

Closeness and situational attribution both resembled collectivism in showing an influence of vertical cultural transmission indicators (in particular the models PARENTSBIRTH and VERTICAL), while dispositional attribution was influenced primarily by participant’s country of birth (model COUNTRYBIRTH). We might predict closeness and situational attribution to change relatively slowly, much like collectivism, while dispositional attribution to show faster cultural change, much like individualism. This again can explain the broader group differences, such as closeness (Fig 1B), which showed little difference between 1^st^ and 2^nd^ generation British Bangladeshis consistent with the strong support for models PARENTSBIRTH and VERTICAL.

The exploratory analyses reinforced several aspects of the model comparison, e.g. that media variables predicted individualism and family contact predicted collectivism. Several measures also showed unanticipated interactions. Closeness showed a culture x age interaction (Fig 2A): British Bangladeshis became closer to others with age, while non-migrants became more distant, perhaps reflecting traditional family-based social support for older family members in the British Bangladeshi community versus state support for older family members in mainstream British society. Younger 1^st^ generation British Bangladeshis may also lack the social ties of older 1^st^ generation migrants who have now established families in the UK. Self-enhancement showed an effect of gender and education (Fig 2B). Men of all cultural backgrounds and education showed unrealistic self-enhancement, with most rating themselves above average on various desirable traits. Non-migrant and 2^nd^-generation British Bangladeshi women of low education rated themselves below average, perhaps reflecting persistent gender stereotypes within the UK, and self-enhancement increased with education. This may attest to the power of education for increasing women’s self-esteem, or indicate that self-confident women are more likely to seek educational opportunities. The landscape drawing task, despite previously shown to be a marker of analytic/holistic cognition [35], here failed to show cultural differences, nor any model support in the model comparison analysis, nor any significant predictors in the exploratory regression analyses. It may be that the drawing task in [35] only measures analytic-holistic cognitive style in societies which have a relevant artistic tradition; while this may be the case in East Asia as shown in [35], it may not be in Bangladesh.

Our study has several limitations. While we aimed to select psychological measures that tap a broad range of psychological processes (both social orientation and analytic-holistic cognition) and that used a range of methods (both verbal Likert-response questions and non-verbal tasks such as categorising objects or drawing landscapes), others may provide better insights into the transmission dynamics of acculturation. Our transmission indicators (e.g. media use) are relatively crude, and vulnerable to self-report and/or memory biases. It would be particularly useful to obtain more detailed and behaviourally-validated interaction frequencies with members of similar and different cultural groups, going beyond our crude self-report measures such as ‘family contact’ and ‘family interaction’. We did not, for example, measure non-familial cultural transmission of heritage values from sources such as religious or community leaders, who may be just as important conduits for maintaining heritage thinking styles as family members. Indeed, such second generation sources may emphasise traditional values or extreme religious or social values to a greater extent than first generation migrants. Socio-economic status was only obtainable from a subset of our sample, and while this was included in the exploratory regression analysis (indicating its importance to individualism and dispositional attribution but not the other measures), it would have been desirable to have accounted for SES differences in the model comparison as well (this was not possible here because model comparison requires all models to be run on the same sized dataset). It would also be useful to know the psychological profiles of the Bangladeshi population from which our participants migrated (although for the older 1^st^ generation participants who migrated decades ago, this is of course impossible given the many changes to have occurred in Bangladesh since the 1970s). Some findings, such as the marginally higher individualism in 1^st^ generation British Bangladeshis compared to non-migrants, might represent the qualities of a self-selected sample who chose to, or were able to, leave Bangladesh. As noted above, however, our findings of high collectivism and broadly similar individualism are consistent with data from Pakistan [32], a country with regional and cultural similarities to Bangladesh, and this increases confidence in our findings. It would also be worth exploring the lability of the psychological processes that we measured here given previous findings of bicultural frame switching in immigrant individuals [43]. British Bangladeshis, like other migrant individuals, may be able to switch between more or less WEIRD thinking styles depending on context (e.g. whether at home with relatives or at work or university). Obtaining the task measures in different contexts, or following different social primes, might yield different results. Finally, we made only limited use of model comparison techniques; we anticipate future researchers designing questionnaire and observational measures both based on our exploratory findings reported here, and more explicitly tied to cultural transmission models [16,17], tapping mechanisms such as conformist or prestige-biased transmission, going beyond our HORIZONTAL and VERTICAL models.

In conclusion, our finding of rapid one-generation psychological acculturation contributes to a growing literature in the evolutionary sciences that views our species as being unusually good at rapidly adapting to local conditions via cultural, rather than genetic, evolution [44–49], and points to the need to understand the cultural transmission dynamics underlying patterns of population-level cultural change [18,19]. Our findings also have important social implications, particularly in these times of increasing international migration. Rapid psychological acculturation belies common fears that immigrant communities—even large and culturally cohesive communities such as London-based British Bangladeshis—will fail to integrate with wider society due to fundamental differences in ways of thinking. At the same time, the loss of certain heritage cultural values, such as close social ties, may be worth actively preserving in the face of their steady decline. In sum, a better understanding of intergenerational psychological change in migrants can provide valuable theoretical and applied insights of use to cultural researchers across anthropological, psychological and biological sciences, as well as policymakers.

## Supporting Information

S1 File Full experimental materials. (PDF)

S2 File Full data set in csv format. (CSV)

S3 File R scripts for all analyses. (R)

S1 Table Full details of model comparisons. (DOCX)

S2 Table Full details of exploratory regression models. (DOCX)
