# Correction: How Do People Become W.E.I.R.D.? Migration Reveals the Cultural Transmission Mechanisms Underlying Variation in Psychological Processes

**DOI:** 10.1371/journal.pone.0203250

**Published:** 2018-08-27

**Authors:** Alex Mesoudi, Kesson Magid, Delwar Hussain

There is an error in Table 4. Table 4 contains a row for the measure “Closeness”. The mean (standard deviation) value of “4.18 (1.79)” for the column “1st gen” and “4.93 (1.87)” for the column “Non-migrants” are swapped. The Non-migrants column should be “4.18 (1.79)” and the 1^st^ gen column should be “4.93 (1.87).”

**Table 4:** Summary of culture-only means and regression coefficients. Means and standard deviations are raw values before transformation. Unstandardised regression coefficients estimate the difference denoted in the column heading, with 95% confidence intervals in square brackets. Note that in the regressions several measures are logged, and some models are non-linear (see text for details), so coefficients should not be compared across models/measures. Differences comprising CIs that do not cross zero are shown in bold. 1st gen = 1st generation British Bangladeshi, 2nd gen = 2nd generation British Bangladeshi. For categorisation, higher values indicate holistic cognition, lower indicate analytic.

Measure	Mean (sd), before transformation			Unstandardised regression coefficients [95% CI] on group differences		
	1^st^ gen	2^nd^ gen	Non-migrants	1^st^ gen - 2^nd^ gen	1^st^ gen–non-migrants	2^nd^ gen–non-migrants
Individualism	5.25 (0.82)	5.13 (0.83)	4.98 (0.95)	0.05 [-0.06, 0.15]	0.08 [-0.02, 0.19]	0.04 [-0.07, 0.15]
Collectivism	6.29 (0.57)	5.93 (0.66)	5.45 (0.77)	**0.19 [0.08, 0.30]**	**0.41 [0.30, 0.51]**	**0.22 [0.11, 0.33]**
Closeness	4.93 (1.87)	4.84 (1.44)	4.18 (1.79)	0.19 [-0.42, 0.79]	**0.80 [0.21, 1.40]**	**0.62 [0.01, 1.23]**
Self-enhancement	37.92 (13.97)	40.86 (17.70)	38.14 (15.48)	-2.72 [-8.22, 2.77]	0.22 [-4.95, 5.38]	2.94 [-2.65, 8.53]
Categorisation (holistic)	0.83 (0.27)	0.82 (0.27)	0.76 (0.29)	0.06 [-0.58, 0.69]	0.41 [-0.15, 0.98]	0.36 [-0.26, 0.97]
Dispositional attribution	4.94 (1.01)	5.14 (0.79)	5.27 (0.88)	-0.19 [-0.51, 0.13]	**-0.33 [-0.62, -0.03]**	-0.13 [-0.46, 0.19]
Situational attribution	5.16 (1.08)	4.87 (1.04)	4.38 (1.01)	0.29 [-0.08, 0.65]	**0.77 [0.43, 1.12]**	**0.48 [0.11, 0.86]**
Drawing task: Horizon ratio	0.59 (0.22)	0.59 (0.18)	0.60 (0.18)	0.01 [-0.07, 0.08]	-0.004 [-0.07, 0.06]	-0.01 [-0.09, 0.06]
Drawing task: Objects	15.43 (24.95)	11.91 (11.39)	14.38 (18.98)	0.02 [-0.36, 0.41]	-0.03 [-0.40, 0.33]	-0.06 [-0.45, 0.33]
