# Supplementary material for: How Do People Become W.E.I.R.D.? Migration Reveals the Cultural Transmission Mechanisms Underlying Variation in Psychological Processes
Source: PLoS One. 2016 Jan 13;11(1):e0147162. doi: 10.1371/journal.pone.0147162 (PMC4711941; doi:10.1371/journal.pone.0147162)
Supplement: S1 File — (PDF) [file pone.0147162.s001.pdf]

INFORMATION SHEET

Title of project: Variation in thinking style

Name and address of investigators: Dr Delwar Hussain and Dr Alex Mesoudi, Department of Anthropology, Durham University, South Road, Durham, DH1 3LE

Contact details of investigator: delwar.hussain@durham.ac.uk

Invitation to take part in this research:
We would like to invite you to participate in this research project. Only participate if you want to; choosing not to take part will not disadvantage you in any way. Before you decide whether you want to take part, it is important for you to understand why the research is being done and what your participation will involve. Please take time to read the following information carefully and discuss it with others if you wish. Ask us if there is anything that is not clear or if you would like more information.

Project description:
We are conducting a research study looking at variation in individuals' thinking styles. You will be asked to complete some short tests, some of which are questionnaires and some simple drawing tasks, followed by some brief questions about yourself (e.g. age, occupation).

Overall, the entire study should last approximately 30 minutes, and you will be compensated £5 for your time.

This study has been fully approved by the Research Ethics and Data Protection Committee of the Department of Anthropology, Durham University. All information collected will be treated as strictly confidential and handled in accordance with the provisions of the Data Protection Act 1998.

Participation, confidentiality, and withdrawal:
It is up to you to decide whether or not to take part. If you do decide to take part you will be given this information sheet to keep and be asked to sign a consent form. If you decide to take part you are still free to withdraw at any time and without giving a reason. You will be identified in our computers by a unique number, and all this information will be kept strictly anonymous.

Durham University

CONSENT FORM

Title of project: Variation in thinking style

Please complete this form after you have read the Information Sheet and/or listened to an explanation about the research.

- I confirm that I have read the Information Sheet, and any questions have been satisfactorily answered by the investigator
- I understand that if I decide at any time during the research that I no longer wish to participate, I can notify the researchers involved and be withdrawn from it immediately with no penalty
- I consent to the use of my personal information for the purposes of this research study. I understand that such information will be treated as strictly confidential and handled in accordance with the provisions of the Data Protection Act 1998

Participant's Statement:

I, ____ (PRINT YOUR NAME) agree that the research project named above has been explained to me to my satisfaction and I agree to take part in the study

Signed: Date:

Investigator's Statement:

I ____ (PRINT YOUR NAME) confirm that I have explained the nature, demands and any foreseeable risks (where applicable) of the proposed research to the volunteer

Signed: Date:

CONFIRMATION OF PAYMENT

Title of project: Variation in thinking style

Participant's Statement:

I, ____ (PRINT YOUR NAME), confirm

that I have received £5 for completion of the above study.

Signed: Date:

Investigator's Statement:

I, ____ (PRINT YOUR NAME), confirm

that the participant named above has received £5 for completion of this study.

Signed: Date:

Thank-you for agreeing to take part in this study.

There are six parts to the study, followed by a few simple questions about yourself.

Remember that it is not a test, and there are no right or wrong answers to any of the questions.

Please complete it in your own time, but try not to take too long on any one section.

Please turn the page to start the first part.

PART 1 of 6

Please rate how much you agree or disagree with the following statements, from 1 (Strongly Agree) to 7 (Strongly Disagree). For each statement circle one number.

1. It is my duty to take care of my family, even when I have to sacrifice what I want.

1	2	3	4	5	6	7
Strongly agree	Moderately agree	Slightly agree	Neither agree nor disagree	Slightly disagree	Moderately disagree	Strongly disagree

2. Parents and children must stay together as much as possible.

1	2	3	4	5	6	7
Strongly agree	Moderately agree	Slightly agree	Neither agree nor disagree	Slightly disagree	Moderately disagree	Strongly disagree

3. Winning is everything.

1	2	3	4	5	6	7
Strongly agree	Moderately agree	Slightly agree	Neither agree nor disagree	Slightly disagree	Moderately disagree	Strongly disagree

4. When another person does better than I do, I get tense and aroused.

1	2	3	4	5	6	7
Strongly agree	Moderately agree	Slightly agree	Neither agree nor disagree	Slightly disagree	Moderately disagree	Strongly disagree

5. I feel good when I cooperate with others.

1	2	3	4	5	6	7
Strongly agree	Moderately agree	Slightly agree	Neither agree nor disagree	Slightly disagree	Moderately disagree	Strongly disagree

6. The well-being of my coworkers is important to me.

1	2	3	4	5	6	7
Strongly agree	Moderately agree	Slightly agree	Neither agree nor disagree	Slightly disagree	Moderately disagree	Strongly disagree

7. If a coworker gets a prize, I would feel proud.

1	2	3	4	5	6	7
Strongly agree	Moderately agree	Slightly agree	Neither agree nor disagree	Slightly disagree	Moderately disagree	Strongly disagree

8. It is important to me that I respect the decisions made by my groups.

1	2	3	4	5	6	7
Strongly agree	Moderately agree	Slightly agree	Neither agree nor disagree	Slightly disagree	Moderately disagree	Strongly disagree

9. Competition is the law of nature.

1	2	3	4	5	6	7
Strongly agree	Moderately agree	Slightly agree	Neither agree nor disagree	Slightly disagree	Moderately disagree	Strongly disagree

10. I often do "my own thing."

1	2	3	4	5	6	7
Strongly agree	Moderately agree	Slightly agree	Neither agree nor disagree	Slightly disagree	Moderately disagree	Strongly disagree

11. It is important that I do my job better than others.

1	2	3	4	5	6	7
Strongly agree	Moderately agree	Slightly agree	Neither agree nor disagree	Slightly disagree	Moderately disagree	Strongly disagree

12. Family members should stick together, no matter what sacrifices are required.

1	2	3	4	5	6	7
Strongly agree	Moderately agree	Slightly agree	Neither agree nor disagree	Slightly disagree	Moderately disagree	Strongly disagree

13. I'd rather depend on myself than others.

1	2	3	4	5	6	7
Strongly agree	Moderately agree	Slightly agree	Neither agree nor disagree	Slightly disagree	Moderately disagree	Strongly disagree

14. To me, pleasure is spending time with others.

1	2	3	4	5	6	7
Strongly agree	Moderately agree	Slightly agree	Neither agree nor disagree	Slightly disagree	Moderately disagree	Strongly disagree

15. My personal identity, independent of others, is very important to me.

1	2	3	4	5	6	7
Strongly agree	Moderately agree	Slightly agree	Neither agree nor disagree	Slightly disagree	Moderately disagree	Strongly disagree

16. I rely on myself most of the time; I rarely rely on others.

1	2	3	4	5	6	7
Strongly agree	Moderately agree	Slightly agree	Neither agree nor disagree	Slightly disagree	Moderately disagree	Strongly disagree

Please think of the single person with whom you feel you have the closest relationship, the person you are most likely to confide in and seek support from. They could be a family member, a romantic partner or a nonromantic friend.

Question 1: Please select your relationship with this person. Tick only ONE box.

Mother ☐ Father ☐ Brother ☐ Sister ☐

Other family member ☐ (Please specify: ______________________)

Husband/wife ☐ Girlfriend/boyfriend ☐

Same-sex nonromantic friend ☐

Opposite-sex nonromantic friend ☐

None of the above ☐ (Please specify: ___________________)

Question 2: Imagine that the circles below represent you ("Self") and the person you selected above ("Other"). Please draw a circle around which of the 7 pictures best describes the closeness of your relationship. Mark only ONE pair of circles.

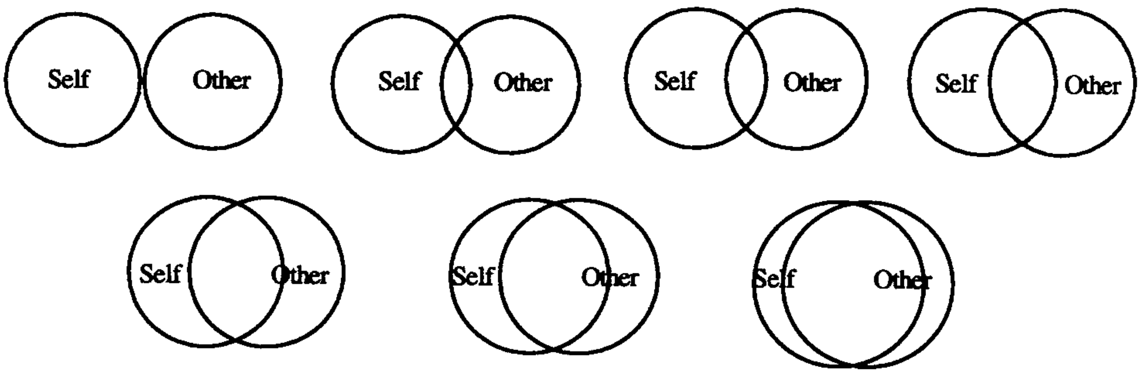

PART 3 of 6

Please estimate what percentage of the UK population, of the same age and gender, who you think are better than you on the following characteristics.

Enter a single number ranging from 0% (= no-one is better than you; you are the best) to 100% (= everyone is better than you; you are the worst).

1. ______ % of the population is more **attractive** than me.

2. ______ % of the population is more **interesting** than me.

3. ______ % of the population is more **cooperative** than me.

4. ______ % of the population is more **intelligent** than me.

5. ______ % of the population is more **loyal** than me.

6. ______ % of the population is more **considerate** than me.

7. ______ % of the population is more **independent** than me.

8. ______ % of the population is more **hard-working** than me.

9. ______ % of the population is more **dependable** than me.

10. _____ % of the population is more **confident** than me.

PART 4 of 6

Printed below are 10 sets of three objects. For each set of three objects, please circle whichever TWO objects you think best go together. Please only circle TWO objects in each row.

1.

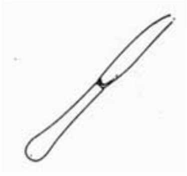 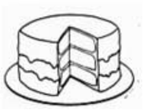 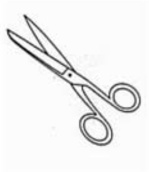

2.

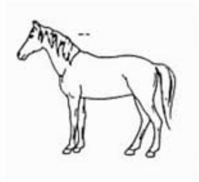 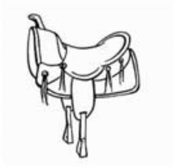 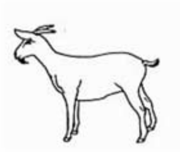

3.

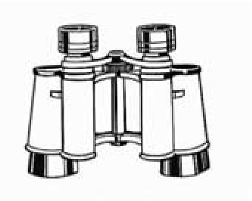 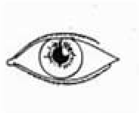 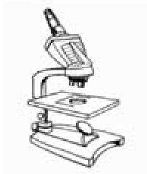

4.

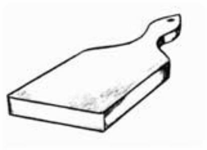 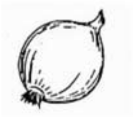 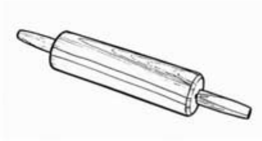

5.

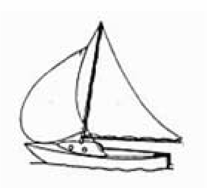 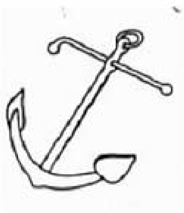 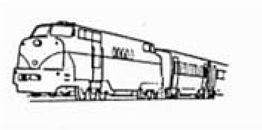

6.

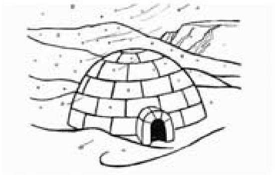 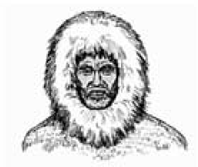 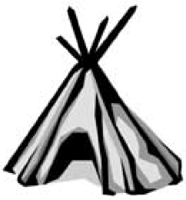

7.

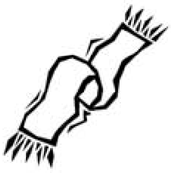 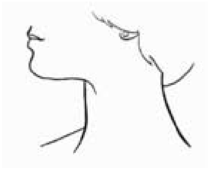 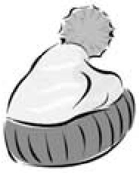

8.

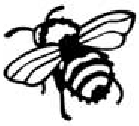 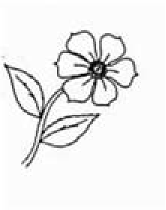 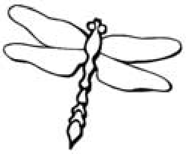

9.

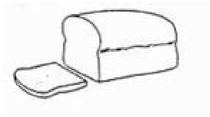 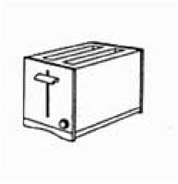 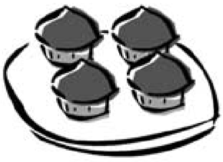

10.

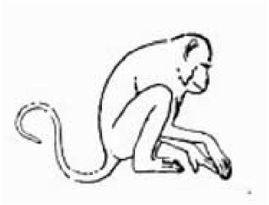 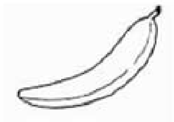 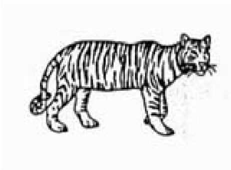

Printed below are short profiles of two notorious real-life people. Please read each description and answer the questions that follow.

Profile 1

Ben Johnson was a leading 100 metres runner in the 1980s. He set 100m world records and won Gold medals at both the 1987 World Championships and the 1988 Summer Olympics. However, these world records and Gold medals were taken back when drug tests revealed that Johnson had been using banned performance-enhancing steroids.

Commentators still debate the reasons why Johnson took banned steroids. Some of these reasons are listed below. For each, please indicate how much you personally think that this factor may have contributed to Johnson's actions. Please note that there is no single correct answer, we are just interested in your personal opinion.

(a) "Johnson took steroids because of his excessive drive to win"

1	2	3	4	5	6	7
Strongly agree	Moderately agree	Slightly agree	Neither agree nor disagree	Slightly disagree	Moderately disagree	Strongly disagree

(b) "Johnson took steroids because athletics had become too competitive"

1	2	3	4	5	6	7
Strongly agree	Moderately agree	Slightly agree	Neither agree nor disagree	Slightly disagree	Moderately disagree	Strongly disagree

(c) "Johnson took steroids because he is naturally a cheating person"

1	2	3	4	5	6	7
Strongly agree	Moderately agree	Slightly agree	Neither agree nor disagree	Slightly disagree	Moderately disagree	Strongly disagree

(d) "Johnson took steroids because they were commonly available to athletes"

1	2	3	4	5	6	7
Strongly agree	Moderately agree	Slightly agree	Neither agree nor disagree	Slightly disagree	Moderately disagree	Strongly disagree

Profile 2

Gang Lu was a Chinese physics student at the University of Iowa who had recently lost an award competition, unsuccessfully appealed it, and subsequently failed to get an academic job. On 31st October 1991, he entered the University of Iowa Physics Department and shot his supervisor, the person who handled his appeal, several fellow students and bystanders, and then himself.

Nobody really knows why Lu committed those terrible acts. Some possible reasons are listed below. For each, please indicate how much you personally think that this factor may have contributed to Lu's actions. Please note that there is no single correct answer, we are just interested in your personal opinion.

(a) "Lu was influenced by American movies and television shows which glorify violent revenge tactics"

1	2	3	4	5	6	7
Strongly agree	Moderately agree	Slightly agree	Neither agree nor disagree	Slightly disagree	Moderately disagree	Strongly disagree

(b) "Lu must have had some kind of mental illness that drove him to commit such violent acts"

1	2	3	4	5	6	7
Strongly agree	Moderately agree	Slightly agree	Neither agree nor disagree	Slightly disagree	Moderately disagree	Strongly disagree

(c) "Lu had become so obsessed with academic success that he could not handle losing"

1	2	3	4	5	6	7
Strongly agree	Moderately agree	Slightly agree	Neither agree nor disagree	Slightly disagree	Moderately disagree	Strongly disagree

(d) "Lu's supervisor and university did not provide enough support to help him cope with the stresses of academic life in a new country"

1	2	3	4	5	6	7
Strongly agree	Moderately agree	Slightly agree	Neither agree nor disagree	Slightly disagree	Moderately disagree	Strongly disagree

In the box below, please draw a landscape scene. Include in your drawing a house, a tree, a river, a person and a horizon. Feel free to draw additional objects if you want.

Finally, please answer the following questions about yourself:

1. What is your age? ____________________

2. What is your sex?   Male ☐   Female ☐

3. In which country were you born? ___________________________

4. In which country (or countries) were your parents born?

   Mother: ___________________ Father: ___________________

5. If you have lived in more than one country in your lifetime, please list those countries and give the age you were when you moved to each country

   ____

6. What is your current occupation? (If you are retired, please give your most recent occupation at the time of retirement)

   ____

7. What are your parents' current or (if retired) most recent occupations?

   Mother: ___________________ Father: ___________________

8. In total, how many years of your life have you spent in formal education (at school, college or university)?

   ____

9. What is the highest educational qualification you have received (e.g. school leaving certificate, GCSE, A Level, BSc, BA)?

   ____

10. Please list below all of the languages that you speak fluently.

____

11. If you listed more than one language in Question 10:
In the table below, please indicate the language(s) that you use to communicate with the person/people listed, and mark with a tick how often you use that language with that person/people (from 1=hardly ever, to 5=always). Leave blank those that do not apply to you.

**Person/people**	**Language(s)**	**How often?** Hardly ever 1	2	3	4	Always 5
Mother						
Father						
Brothers/ sisters						
Grandparents						
Grandparents' generation						
Parents' generation						
Husband/wife						
Friends						
Children						
During your early life						
Work colleagues						
Counting						
Writing						
At school / university						
Grandchildren						

12. In an average week, how many family members (including extended family such as cousins and grandparents) do you see in person?

____

13. In an average week, how many family members (including extended family such as cousins and grandparents) do you communicate with by phone or email, but don't see in person?

____

14. In the table below, please list up to five family members who you interact with **most** during an average week. For each person, please specify roughly how many hours per week that you spend with them.

Relative (e.g. “mother”, “father”, “brother”)	On average, how many hours per week do you spend with this person?

15. In the table below, please list up to five people who you interact with most during an average week, **excluding family members**. To maintain anonymity, identify them using an initial (e.g. “P.”). For each person, please provide the information requested.

Person (use initials to keep them anonymous)	Relationship (e.g. “work colleague”, “neighbour”, “university friend”)	On average, how many hours per week do you spend with this person?	In which country was this person born and raised?	In which country was this person's parents born and raised?

16. On average, how often do you read a UK-based daily newspaper?

Every day ☐ At least once a week ☐

At least once a month ☐ Less than once a month ☐

17. On average, how often do you read a UK-based magazine?

Every day ☐ At least once a week ☐

At least once a month ☐ Less than once a month ☐

18. In an average day, how many hours do you spend watching UK-based television channels (e.g. BBC, ITV, Sky)?

____

19. In an average day, how many hours do you spend on the internet?

____

20. What religion (e.g. Catholicism, Islam, Hinduism) would you describe yourself as a member of, if any?

____

21. To what extent, on the scale of 1-7 below, would you describe yourself as a religious person? Please circle one number.

1	2	3	4	5	6	7
Extremely religious, religion affects every aspect of my life	Very religious	Moderately religious	Quite religious	Slightly religious	Not very religious	Not at all religious, religion does not affect me at all

22. Finally, please only answer this question if you or your parents were **not** born in the United Kingdom.

The country where you or your parents grew up, if different from where you live now (the UK), is sometimes called your *heritage culture*. For example, if your parents were born and raised in Bangladesh, then 'Bengali' might be your heritage culture. Or if you grew up in Korea and then moved to the UK, then 'Korean' would be your heritage culture. In the space below, please give your heritage culture. If there are several cultures in your family background, pick the one that influenced you most.
My heritage culture is ________________________________

Please circle ONE of the numbers to the right of each statement to indicate your level of agreement or disagreement.

	**Agree**						**Disagree**
I often participate in my heritage cultural traditions.	1	2	3	4	5	6	7
I often participate in mainstream British cultural traditions.	1	2	3	4	5	6	7
I would be willing to marry a person from my heritage culture.	1	2	3	4	5	6	7
I would be willing to marry a British person who is not from my own heritage culture.	1	2	3	4	5	6	7
I enjoy social activities with people from the same heritage culture as myself.	1	2	3	4	5	6	7
I enjoy social activities with British people who are not from my own heritage culture.	1	2	3	4	5	6	7
I am comfortable interacting with people of the same heritage culture as myself.	1	2	3	4	5	6	7
I am comfortable interacting with British people who are not from my own heritage culture.	1	2	3	4	5	6	7
I enjoy entertainment (e.g. movies, music) from my heritage culture.	1	2	3	4	5	6	7
I enjoy British entertainment (e.g. movies, music).	1	2	3	4	5	6	7
I often behave in ways that are typical of my heritage culture.	1	2	3	4	5	6	7
I often behave in ways that are typical for a British person not from my heritage culture.	1	2	3	4	5	6	7
It is important for me to maintain or develop the practices of my heritage culture.	1	2	3	4	5	6	7
It is important for me to maintain or develop mainstream British cultural practices.	1	2	3	4	5	6	7
I believe in the values of my heritage culture.	1	2	3	4	5	6	7
I believe in mainstream British values.	1	2	3	4	5	6	7
I enjoy the jokes and humour of my heritage culture.	1	2	3	4	5	6	7
I enjoy mainstream British jokes and humor.	1	2	3	4	5	6	7
I am interested in having friends from my heritage culture.	1	2	3	4	5	6	7
I am interested in having British friends who are not from my heritage culture.	1	2	3	4	5	6	7

END OF STUDY
THANK-YOU FOR PARTICIPATING
