# Supplementary material for: How Do People Become W.E.I.R.D.? Migration Reveals the Cultural Transmission Mechanisms Underlying Variation in Psychological Processes
Source: PLoS One. 2016 Jan 13;11(1):e0147162. doi: 10.1371/journal.pone.0147162 (PMC4711941; doi:10.1371/journal.pone.0147162)
Supplement: S1 Table — (DOCX) [file pone.0147162.s004.docx]

**Supplementary S1 Table for**

**How do people become W.E.I.R.D.? Migration reveals the cultural transmission mechanisms underlying variation in psychological processes**

Alex Mesoudi^1,2^, Kesson Magid^2^, Delwar Hussain^3^

^1^ Human Biological and Cultural Evolution Group, Department of Biosciences, University of Exeter, UK

^2^ Department of Anthropology, Durham University, UK

^3^ School of Social and Political Science, University of Edinburgh, UK

Corresponding author details:

Alex Mesoudi, Human Biological and Cultural Evolution Group, Department of Biosciences, College of Life and Environmental Sciences, University of Exeter Cornwall Campus, Penryn, Cornwall TR10 9FE, United Kingdom

Email: [a.mesoudi@exeter.ac.uk](mailto:a.mesoudi@exeter.ac.uk)

**S1 Table. Full details of model comparisons**

| **Measure (global model fit)** | **Model** | **K** | **logLik** | **AIC_c_** | **∆_i_** | **ω_i_** |
| --- | --- | --- | --- | --- | --- | --- |
| Individualism (F(12,242)=1.89, p=.036) | HCT | 9 | -49.83 | 118.40 | 0.00 | 0.80 |
|  | DEM | 4 | -57.43 | 123.02 | 4.63 | 0.08 |
|  | PAR | 5 | -56.99 | 124.23 | 5.83 | 0.04 |
|  | BIR | 5 | -57.04 | 124.32 | 5.92 | 0.04 |
|  | CUL | 6 | -56.92 | 126.18 | 7.78 | 0.02 |
|  | GLB | 14 | -48.51 | 126.77 | 8.38 | 0.01 |
|  | VCT | 9 | -55.78 | 130.29 | 11.89 | 0.00 |
| Collectivism (F(12,241)=8.64, p<.001) | CUL | 6 | -63.02 | 138.38 | 0.00 | 0.58 |
|  | GLB | 14 | -55.16 | 140.08 | 1.70 | 0.25 |
|  | VCT | 9 | -61.11 | 140.95 | 2.57 | 0.16 |
|  | PAR | 5 | -68.37 | 146.97 | 8.59 | 0.01 |
|  | BIR | 5 | -71.74 | 153.72 | 15.34 | 0.00 |
|  | HCT | 9 | -70.02 | 158.77 | 20.39 | 0.00 |
|  | DEM | 4 | -96.38 | 200.93 | 62.55 | 0.00 |
| Closeness (Χ^2^(12)=19.60, p=.075) | PAR | 9 | -476.53 | 971.79 | 0.00 | 0.47 |
|  | VCT | 13 | -472.69 | 972.87 | 1.08 | 0.27 |
|  | CUL | 10 | -476.39 | 973.68 | 1.90 | 0.18 |
|  | BIR | 9 | -478.71 | 976.15 | 4.36 | 0.05 |
|  | DEM | 8 | -480.92 | 978.42 | 6.64 | 0.02 |
|  | GLB | 18 | -471.37 | 981.61 | 9.82 | 0.00 |
|  | HCT | 13 | -477.24 | 981.97 | 10.18 | 0.00 |
| Self enhancement (F(12,243)=0.87, p=.58) | Poor global model fit | | | | | |
| Categorisation (F(12,244)=0.75, p=.70) | Poor global model fit | | | | | |
| Dispositional attribution (F(12,244)=2.17, p=.01) | BIR | 5 | -338.82 | 687.88 | 0.00 | 0.41 |
|  | CUL | 6 | -338.37 | 689.08 | 1.20 | 0.23 |
|  | VCT | 9 | -335.53 | 689.78 | 1.90 | 0.16 |
|  | PAR | 5 | -340.07 | 690.39 | 2.51 | 0.12 |
|  | HCT | 9 | -336.50 | 691.74 | 3.86 | 0.06 |
|  | GLB | 14 | -332.28 | 694.29 | 6.41 | 0.02 |
|  | DEM | 4 | -343.52 | 695.21 | 7.33 | 0.01 |
| Situational attribution (F(12,244)=3.08, p<.001) | CUL | 6 | -371.74 | 755.82 | 0.00 | 0.53 |
|  | PAR | 5 | -372.99 | 756.22 | 0.40 | 0.43 |
|  | BIR | 5 | -375.87 | 761.98 | 6.16 | 0.02 |
|  | VCT | 9 | -372.97 | 764.67 | 8.85 | 0.01 |
|  | HCT | 9 | -373.59 | 765.92 | 10.09 | 0.00 |
|  | GLB | 14 | -368.81 | 767.36 | 11.54 | 0.00 |
|  | DEM | 4 | -384.50 | 777.15 | 21.33 | 0.00 |
| Horizon ratio (F(12,210)=0.47, p=.93) | Poor global model fit | | | | | |
| Additional objects (Χ^2^(12)=11.18, p=.514) | Poor global model fit | | | | | |
