# Supplementary material for: How Do People Become W.E.I.R.D.? Migration Reveals the Cultural Transmission Mechanisms Underlying Variation in Psychological Processes
Source: PLoS One. 2016 Jan 13;11(1):e0147162. doi: 10.1371/journal.pone.0147162 (PMC4711941; doi:10.1371/journal.pone.0147162)
Supplement: S2 Table — (DOCX) [file pone.0147162.s005.docx]

**Supplementary S2 Table for**

**How do people become W.E.I.R.D.? Migration reveals the cultural transmission mechanisms underlying variation in psychological processes**

Alex Mesoudi^1,2^, Kesson Magid^2^, Delwar Hussain^3^

^1^ Human Biological and Cultural Evolution Group, Department of Biosciences, University of Exeter, UK

^2^ Department of Anthropology, Durham University, UK

^3^ School of Social and Political Science, University of Edinburgh, UK

Corresponding author details:

Alex Mesoudi, Human Biological and Cultural Evolution Group, Department of Biosciences, College of Life and Environmental Sciences, University of Exeter Cornwall Campus, Penryn, Cornwall TR10 9FE, United Kingdom

Email: [a.mesoudi@exeter.ac.uk](mailto:a.mesoudi@exeter.ac.uk)

**S2 Table. Full details of exploratory regression models**

| **Measure** | **Predictor** | **B** | **SE** | **t or z value** | **p** |
| --- | --- | --- | --- | --- | --- |
| Individualism (all participants) | Intercept | .522 | .088 | 5.92 | <.001*** |
|  | 2^nd^ gen (vs non-migrant) | .060 | .052 | 1.16 | .247 |
|  | 1^st^ gen (vs non-migrant) | .158 | .053 | 2.99 | .003** |
|  | Occupation: tertiary (vs school) | -.037 | .048 | 0.77 | .443 |
|  | Occupation: graduate (vs school) | .133 | .062 | 2.13 | .034* |
|  | Print media | .130 | .028 | 4.65 | <.001*** |
| Individualism (1-BB & 2-BB only) | Intercept | .124 | .298 | 0.42 | .679 |
|  | 1^st^ gen (vs 2^nd^ gen) | .030 | .101 | 0.30 | .765 |
|  | Occupation: tertiary (vs school) | -.093 | .092 | 1.00 | .322 |
|  | Occupation: graduate-level (vs school) | .103 | .112 | 0.92 | .362 |
|  | Print media | .218 | .060 | 3.61 | <.001*** |
|  | UK acculturation | -.125 | .047 | 2.69 | .010** |
|  | Heritage acculturation | .157 | .052 | 3.03 | .004** |
| Collectivism (all participants) | Intercept | .924 | .044 | 20.91 | <.001*** |
|  | 2^nd^ gen (vs non-migrant) | .087 | .059 | 1.47 | .142 |
|  | 1^st^ gen (vs non-migrant) | .279 | .061 | 4.54 | .001*** |
|  | Female (vs male) | .076 | .038 | 2.00 | .047* |
|  | Religiosity | .031 | .015 | 2.02 | .044* |
|  | Family contact | .010 | .005 | 2.52 | .025* |
| Collectivism (1-BB & 2-BB only) | Intercept | .451 | .218 | 2.07 | .043* |
|  | 1^st^ gen (vs 2^nd^ gen) | .046 | .081 | 0.57 | .570 |
|  | Female (vs male) | .028 | .080 | 0.35 | .731 |
|  | Religiosity | -.004 | .032 | -0.12 | .904 |
|  | Family contact | .024 | .024 | 2.61 | .011* |
|  | Heritage acculturation | .141 | .036 | 3.97 | .001*** |
| Collectivism (1-BB only) | Intercept | .682 | .423 | 1.62 | .119 |
|  | Female (vs male) | -.050 | .112 | -0.44 | .661 |
|  | Religiosity | .057 | .050 | 1.13 | .270 |
|  | Family contact | .010 | .021 | 0.46 | .647 |
|  | Heritage acculturation | .020 | .063 | 0.31 | .757 |
|  | Age of migration | .013 | .006 | 2.29 | .031* |
| Closeness | 2^nd^ gen (vs non-migrant) | -1.00 | 0.77 | 1.30 | .195 |
|  | 1^st^ gen (vs non-migrant) | -2.19 | 0.76 | 2.89 | .004** |
|  | Age | -0.03 | 0.01 | 2.52 | .012* |
|  | 2^nd^ gen x age | 0.05 | 0.03 | 2.07 | .038* |
|  | 1^st^ gen x age | 0.08 | 0.02 | 4.16 | <.001*** |
| Self-enhancement | Intercept | 39.99 | 9.13 | 4.38 | <.001*** |
|  | 2^nd^ gen (vs non-migrants) | -8.68 | 13.89 | 0.63 | .533 |
|  | 1^st^ gen (vs non-migrants) | 1.45 | 10.82 | 0.13 | .893 |
|  | Female (vs male) | 32.64 | 12.90 | 2.53 | .012* |
|  | Years of education | -0.27 | 0.55 | 0.50 | .621 |
|  | 2^nd^ gen x female | -2.28 | 19.51 | 0.12 | .907 |
|  | 1^st^ gen x female | -36.51 | 15.06 | 2.42 | .016* |
|  | 2^nd^ gen x years of education | 0.61 | 0.82 | 0.75 | .453 |
|  | 1^st^ gen x years of education | 0.12 | 0.66 | 0.18 | .860 |
|  | Female x years of education | -1.72 | 0.77 | 2.24 | .026* |
|  | 2^nd^ gen x female x years of education | 0.44 | 1.14 | 0.38 | .702 |
|  | 1^st^ gen x female x years of education | 1.87 | 0.92 | 2.03 | .043* |
| Categorisation | Intercept | 1.53 | 0.33 | 4.69 | <0.001*** |
|  | 2^nd^ gen (vs non-migrant) | 0.56 | 0.29 | 1.91 | .058 |
|  | 1^st^ gen (vs non-migrant) | 0.76 | 0.32 | 2.37 | .019* |
|  | Occupation: tertiary (vs school) | 0.10 | 0.29 | 0.35 | .725 |
|  | Occupation: graduate (vs school) | -0.70 | 0.34 | 2.06 | .041* |
|  | TV viewing | -0.10 | 0.05 | 2.03 | .044* |
| Dispositional attribution | Intercept | 4.61 | 0.24 | 19.27 | <.001*** |
|  | 2^nd^ gen (vs non-migrant) | 0.05 | 0.15 | 0.36 | .721 |
|  | 1^st^ gen (vs non-migrant) | -0.26 | 0.15 | 1.74 | .084 |
|  | Age | 0.01 | 0.01 | 2.41 | .017* |
|  | Occupation: tertiary (vs school) | 0.20 | 0.14 | 1.44 | .152 |
|  | Occupation: graduate (vs school) | 0.46 | 0.18 | 2.60 | .010* |
| Situational attribution | Intercept | 3.62 | 0.32 | 11.29 | <.001*** |
|  | 2^nd^ gen (vs non-migrant) | 2.03 | 0.65 | 3.13 | .002** |
|  | 1^st^ gen (vs non-migrant) | 1.79 | 0.40 | 4.43 | <.001*** |
|  | Languages spoken | 0.68 | 0.27 | 2.51 | .013* |
|  | 2^nd^ gen x languages | -1.07 | 0.39 | 2.78 | .006** |
|  | 1^st^ gen x languages | -0.80 | 0.29 | 2.75 | .006** |
